# Interaction of the TNFR-Receptor Associated Factor TRAF1 with I-Kappa B Kinase-2 and TRAF2 Indicates a Regulatory Function for NF-Kappa B Signaling

**DOI:** 10.1371/journal.pone.0012683

**Published:** 2010-09-13

**Authors:** Kalsoom Sughra, Andreas Birbach, Rainer de Martin, Johannes A. Schmid

**Affiliations:** Department of Vascular Biology and Thrombosis Research, Center for Physiology and Pharmacology, Medical University Vienna, Vienna, Austria; Ohio State University, United States of America

## Abstract

**Background:**

I-kappa B kinase 2 (IKK2 or IKK-beta) is one of the most crucial signaling kinases for activation of NF-kappa B, a transcription factor that is important for inflammation, cell survival and differentiation. Since many NF-kappa B activating pathways converge at the level of IKK2, molecular interactions of this kinase are pivotal for regulation of NF-kappa B signaling.

**Methodology/Principal Findings:**

We searched for proteins interacting with IKK2 using the C-terminal part (amino acids 466–756) as bait in a yeast two-hybrid system and identified the N-terminal part (amino acids 1–228) of the TNF-receptor associated factor TRAF1 as putative interaction partner. The interaction was confirmed in human cells by mammalian two-hybrid and coimmunoprecipitation experiments. The IKK2/TRAF1 interaction seemed weaker than the interaction between TRAF1 and TRAF2, an important activating adapter molecule of NF-kappa B signaling. Reporter gene and kinase assays using ectopic expression of TRAF1 indicated that it can both activate and inhibit IKK2 and NF-kappa B. Co-expression of fluorescently tagged TRAF1 and TRAF2 at different ratios implied that TRAF1 can affect clustering and presumably the activating function of TRAF2 in a dose dependent manner.

**Conclusions/Significance:**

The observation that TRAF1 can either activate or inhibit the NF-kappa B pathway and the fact that it influences the oligomerization of TRAF2 indicates that relative levels of IKK2, TRAF1 and TRAF2 may be important for regulation of NF-kappa B activity. Since TRAF1 is an NF-kappa B induced gene, it might act as a feedback effector molecule.

## Introduction

The NF-kappa B family of transcription factors is essential for a large variety of biological processes such as inflammation, cell survival, regulation of apoptosis, proliferation and cell differentiation. There are two major signaling pathways leading to NF-kappa B: the classical or canonical pathway originating at TNFα-, IL-1 or Toll-like receptors and the alternative pathway initiated for instance at CD40 [1]. Both pathways converge at the level of the IκB kinase (IKK) complex, which contains two related kinases: IKK1 (IKK-alpha) and IKK2 in conjunction with an essential adapter (termed NEMO for NF-kappa B essential modulator, or IKK-gamma). The I-kappa B kinases can then phosphorylate inhibitors of NF-kappa B on two adjacent serine residues, marking them for polyubiquitination, which results in their degradation by 26S proteasomes and release of active NF-kappa B. The classical activation pathway signals primarily to IKK2, whereas the alternative pathway triggers predominantly IKK1 activity [1], [2]. Nevertheless, these two kinases influence each other [3], [4] and interact with a variety of additional signaling molecules [1]. It is currently still not clear, which interactions can occur simultaneously and whether certain molecular associations are mutually exclusive or influence each other, and as a consequence also the NF-kappa B signaling cascade.

In the last few years, it became increasingly clear that ubiquitination processes exert important functions in the activation of the IKK complex [2]. These ubiquitinations are triggered by TRAF molecules (mainly TRAF2, TRAF5 and TRAF6), which contain RING domains that have E3 ligase activity catalyzing non-degradative K63-linked polyubiquitination. In contrast to K48-linked polyubiquitin, K63-linked polyubiquitin chains do not lead to proteasomal degradation but rather serve as an association and signaling platform for certain ubiquitin binding proteins, such as TAB1 and TAB2 in combination with the kinase TAK1 [5]. K63-linked polyubiquitination thereby results in binding and activation of TAK1, which then activates IKK2. TRAF1 is the only TRAF-adapter molecule lacking a RING domain and therefore does not act as a ubiquitin ligase [6], [7]. Of note, TRAF molecules form homo- or heterotrimeric complexes. It has been suggested that the composition of heterotrimers is important for signaling function [8]. Interestingly, both positive and negative regulatory effects of TRAF1 on NF-kappa B signaling have been reported. In cell culture systems overexpression of TRAF1 resulted either in inhibition [9] or in augmentation [10] of NF-kappa B activity. Similar conflicting data have been obtained in knockout mouse models. T-cells from TRAF1-deficient mice showed enhanced IKK2 and NF-kappa B activity [11], whereas dendritic cells from TRAF1-deficient mice showed attenuated NF-kappa B signaling in a different study [12]. The effect of TRAF1 on signaling is further complicated by the fact that it is a substrate of caspases and therefore cleaved in the course of apoptosis. This leads to a release of the TRAF-domain, which then acts as an inhibitor of NF-kappa B signaling [13].

In this study, we provide evidence for a specific interaction of TRAF1 with IKK2 and we demonstrate that this molecular association is weaker than the TRAF1/TRAF2 interaction. Furthermore, we find that ectopic expression of TRAF1 can have both inhibitory and stimulatory effects on IKK2 and NF-kappa B activity. Thus we propose a model in which relative levels of TRAF1, TRAF2 and IKK2 are important for regulating the signaling activity of IKK2.

## Results and Discussion

**Figure 1:**
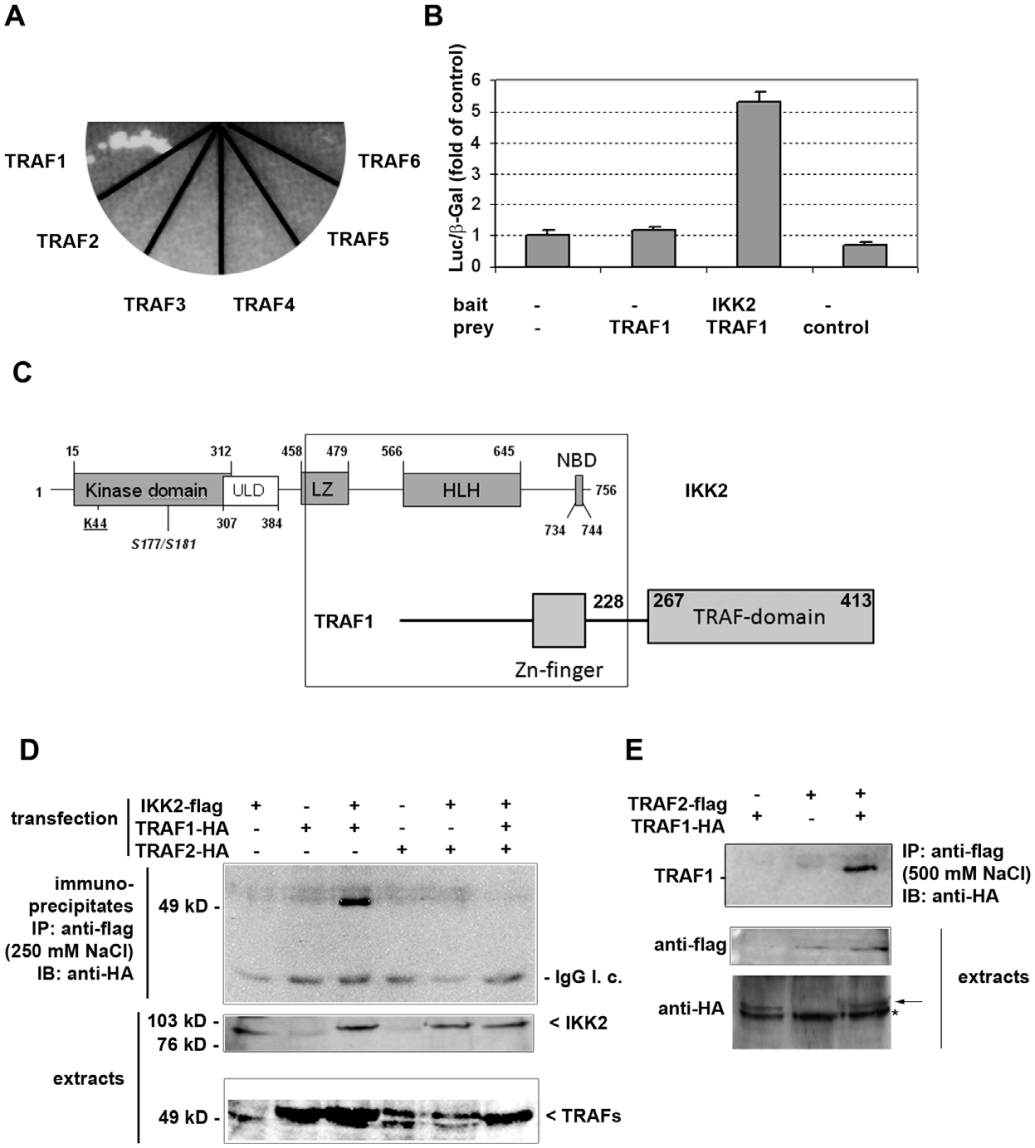
IKK2 interacts specifically with TRAF1. **A**) Yeast two hybrid assay: The C-terminal domain of IKK2 interacts specifically with TRAF1 but not TRAF2 – TRAF6. **B**) Verification of the interaction in mammalian cells using a mammalian two-hybrid system. A luciferase reporter assay was done with empty (-) bait or prey constructs, IKK2, TRAF1 or a control prey (control, pVP16-SV40 large T antigen) as indicated. **C**) Schematic illustration of the interaction domains as depicted by the overlapping rectangle. Amino acids 466–756 of IKK2 including a leucine zipper (LZ), a helix-loop-helix domain (HLH) and the NEMO binding domain (NBD) interact with amino acids 1–228 of TRAF1 (containing a Zn-finger). **D**) Co-immunoprecipitation of IKK2 and TRAF1 after transfection of HeLa cells with flag- or HA-tagged expression constructs as indicated and immunoprecipitation (IP) with flag-affinity matrix in presence of 250 mM NaCl followed by immunoblot (IB) analysis of HA-tagged proteins and IKK2 in immunopreciptates and extracts. **E**) Coimmuno-precipitation of TRAF1 and TRAF2 in presence of 500 mM NaCl analogous to D. The arrow represents the specific HA-TRAF1 band in the extracts; the asterisk denotes an unspecific band.

**Figure 2:**
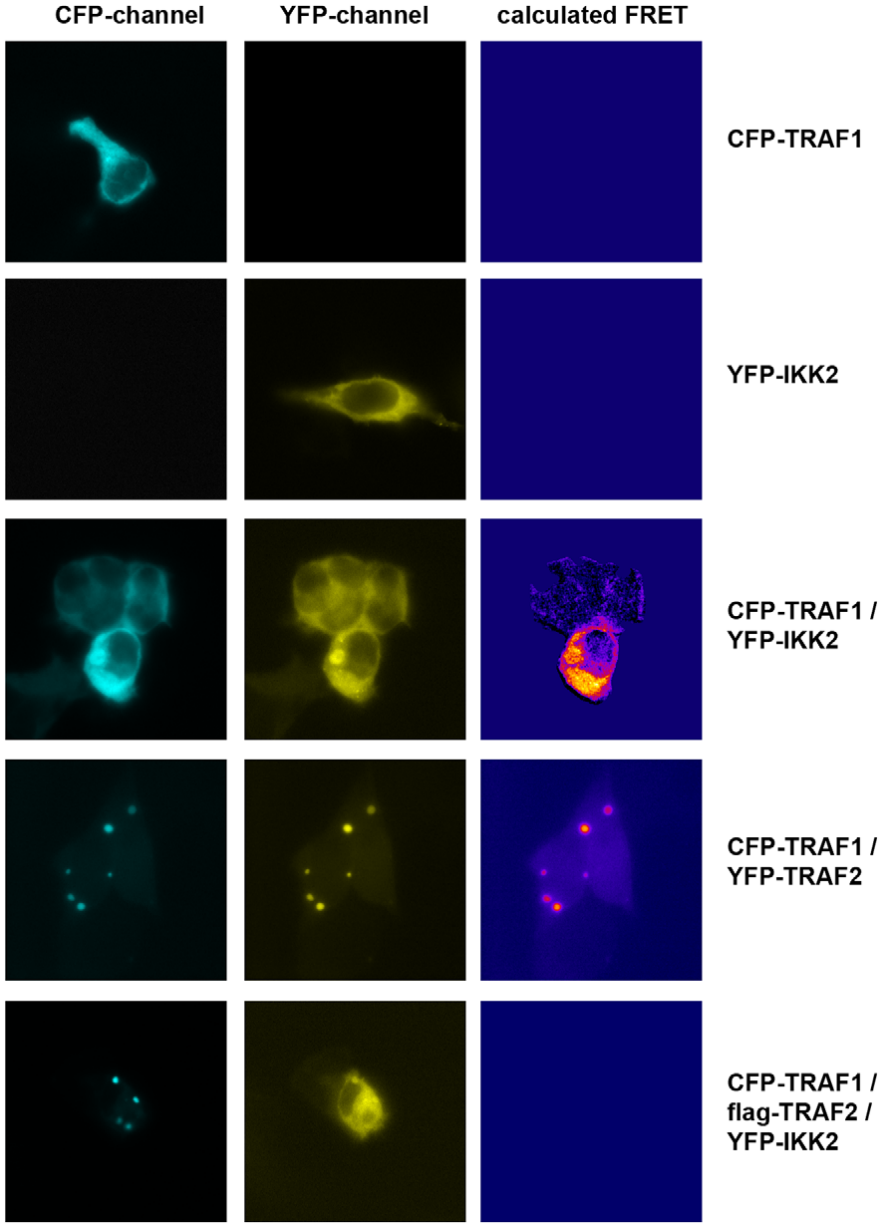
TRAF2 competes with IKK2 for TRAF1 binding. The indicated CFP-, YFP- or flag-tagged expression constructs were transfected into HEK-293 cells followed by FRET-microscopy to determine protein interactions. The signals in the CFP- and YFP-channel are shown, as well as a pseudo-colored calculated FRET images.

**Figure 3:**
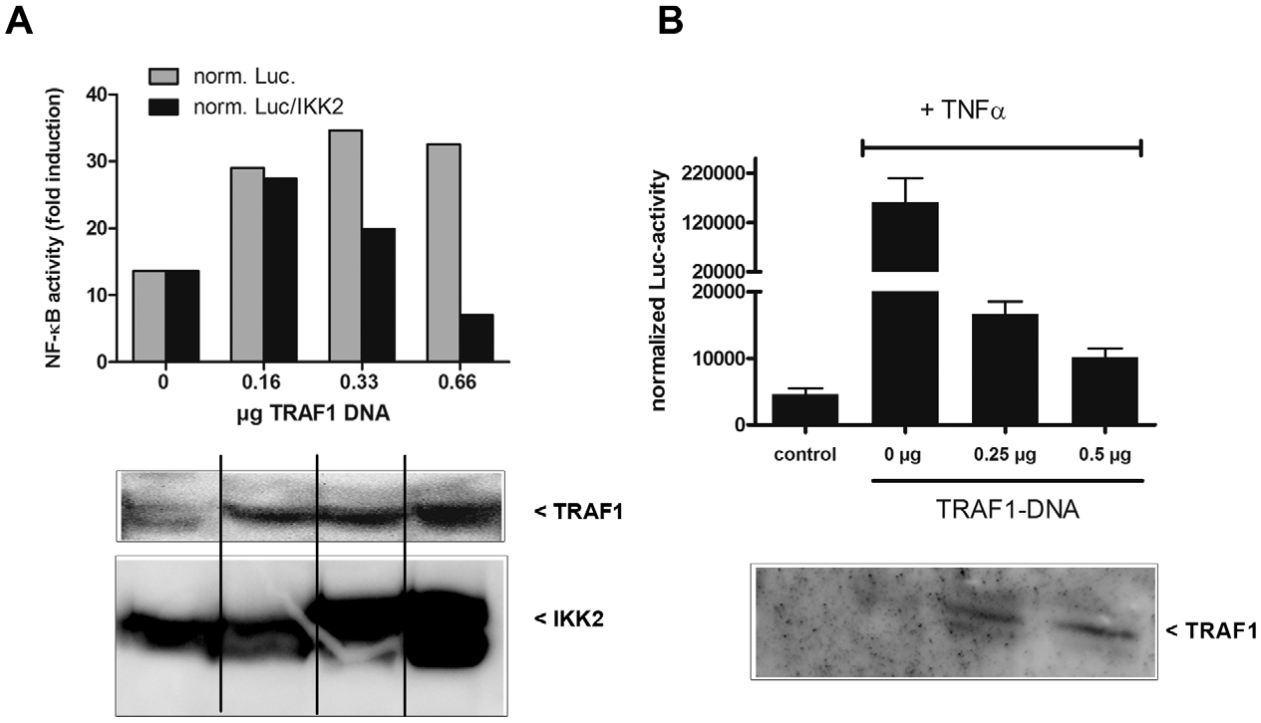
TRAF1 can exert both inhibitory and stimulatory effects on IKK2 and NF-kappa B activity. Effects of TRAF1 in NF-kappa B reporter gene assays: **A**) HEK-293 cells were transfected with a NF-kappa B luciferase reporter and IKK2 in absence or presence of a TRAF1 expression construct at increasing concentrations. Upregulation of NF-kappa B activity by IKK2 is shown as x-fold of a negative control and given as normalized Luciferase activity (norm. Luc.: normalized to constitutive β-Galactosidase expression) or normalized Luciferase activity related to IKK2 protein levels (norm. Luc/IKK2). TRAF1 and IKK2 protein levels are determined by Western Blot analysis of the luciferase extracts. **B**) HeLa cells were transfected with the NF-kappa B reporter and a TRAF1 construct at increasing concentrations followed by treatment with TNFα 50 ng/ml for 6 h) as indicated. NF-kappa B Luciferase activity was determined normalized to constitutive β-Galactosidase expression. Levels of TRAF1 in the extracts have been determined by Western Blot analysis.

**Figure 4:**
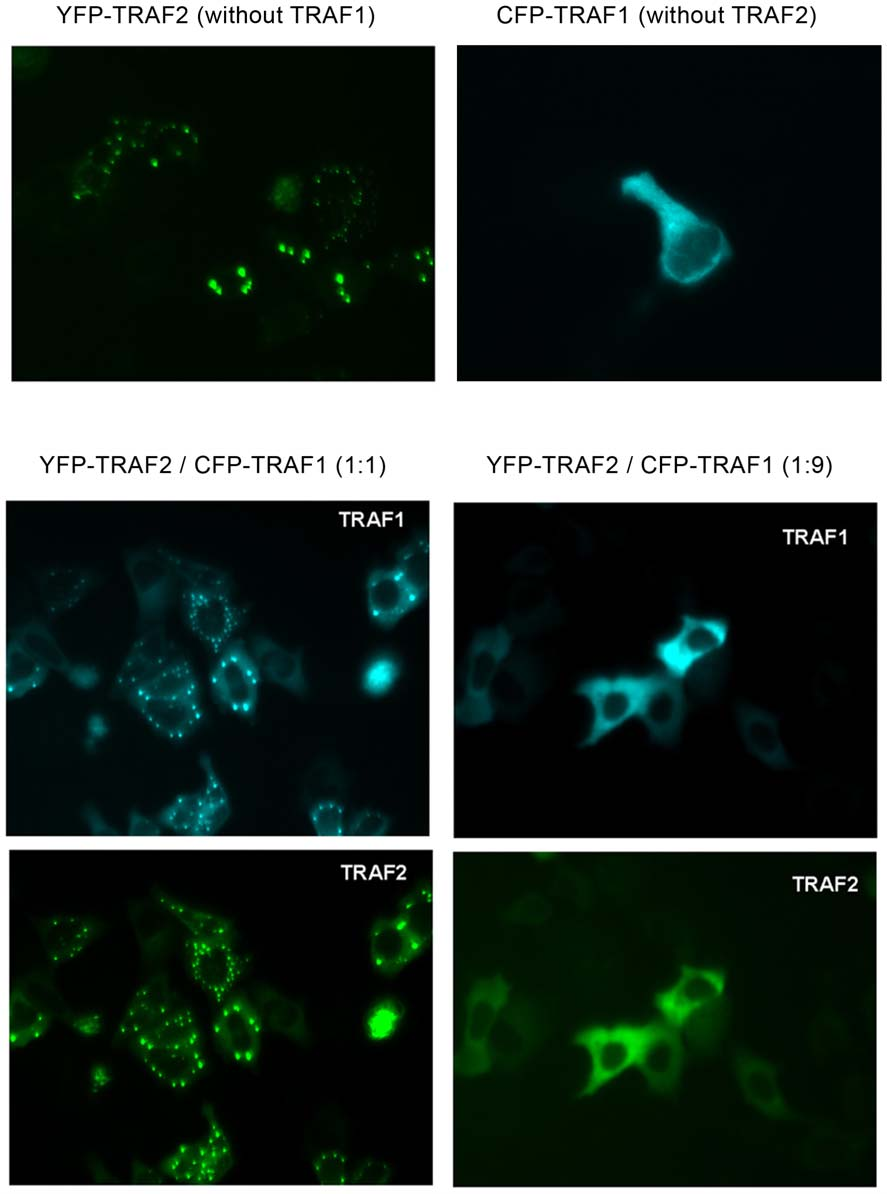
TRAF1 influences the clustering tendency of TRAF2 in a dose dependent manner. YFP-tagged TRAF2 or CFP-tagged TRAF1 were expressed in HEK-293 cells either alone or in combination at a ratio of 1∶1 or 1∶9 as indicated and analyzed by fluorescence microscopy. A higher level of TRAF1 led to the disaggregation of TRAF2 clusters.

Our aim was to identify interaction partners of IKK2, a key enzyme for NF-kappa B activation. To this end, we performed a yeast two-hybrid screen with the C-terminal part of IKK2 as a bait as described [14]. This part contains a helix-loop-helix domain and a leucine zipper as potential protein interaction domains. Among various signaling molecules, we identified an N-terminal fragment of TRAF1 (amino acids 1–228) as a putative binding partner. Next, we tested whether other members of the TRAF family are capable of interacting with the IKK2 C-terminal bait using yeast two-hybrid constructs for all TRAFs. In this system, only TRAF1 interacted with the IKK2-bait (Fig.1A). Testing IKK1 as the bait in combination with all the TRAF molecules (TRAF1–TRAF6) did not reveal any significant interaction (data not shown). The binding of IKK2 and TRAF1 could be verified in a mammalian two-hybrid reporter assay, thus confirming the interaction in mammalian cells (Fig. 1B). Preferential interaction between IKK2 and TRAF1 but not other TRAF family members is supported by the fact that IKK2 interacts with the N-terminal part of TRAF1, which differs from all the other TRAF family members (Fig. 1C). The C-terminal interaction domain of IKK2 does not include the kinase domain, suggesting that it may still be accessible for substrates. Furthermore, we could clearly demonstrate the interaction between full length IKK2 and full-length TRAF1 in human cells by co-immunoprecipitation experiments (Fig. 1D). Similar experiments with the N-terminal part (amino acids 1–228) of TRAF1 verified the results of the yeast two hybrid system that the N-terminal domain lacking the TRAF domain is sufficient for the interaction with IKK2 (data not shown). However, it has to be noted that also the TRAF-domain seems to be capable of interacting with the IKK-complex [13]. In contrast to the co-immunoprecipitation of TRAF1 with IKK2, we could not detect co-precipitation of TRAF2 with IKK2 in our system. This is in contrast to a previous report using a different cell line demonstrating an interaction between IKK2 and TRAF2 [15]. In that report, the TRAF2/IKK2 interaction appeared stronger for a truncated IKK2-construct containing the leucine zipper than for full length IKK2. The study also did not test for interaction of IKK2 with other TRAF family members. In conclusion, we assume that IKK2 has the propensity to interact with both TRAF1 and TRAF2, but that the interaction with TRAF1 seems to be stronger in our experimental system. This is in line with our observation that co-expression of TRAF2 reduced the amount of TRAF1 co-precipitating with IKK2. In this case, TRAF2 may compete with IKK2 for TRAF1 binding (Fig. 1D). This possibility was further tested by co-immunoprecipitation of TRAF1 and TRAF2, which revealed that these two molecules interact with each other at different salt concentrations up to a rather high NaCl concentration of 500 mM (Fig. 1E). At this salt concentration, we could not detect any significant interaction between TRAF1 and IKK2 (data not shown) indicating that TRAF1/TRAF2 binding may be stronger than the association between TRAF1 and IKK2 (which was detectable at a concentration of 250 mM NaCl). Consequently, it has to be expected that TRAF1 would rather bind TRAF2 as long as TRAF2 binding sites are available. This is further supported by microscopy-based interaction studies applying the method of fluorescence resonance energy transfer (FRET). Both the interactions between YFP-tagged IKK2 and CFP-tagged TRAF1 and between CFP-TRAF1 and YFP-TRAF2 could be visualized by FRET microscopy in the cytosol. However, when CFP-TRAF1 and YFP-IKK2 were coexpressed in presence of flag-tagged TRAF2, the interaction between TRAF1 and IKK2 became undetectable and TRAF1 appeared in clusters characteristic for ectopic expression of TRAF2 (Fig. 2). This indicates that TRAF2 competes with IKK2 for binding of TRAF1. Testing the effect of TRAF1 expression in NF-kappa B reporter gene assays revealed either up- or downregulation of NF-kappa B activity dependent on the stimulus and the expression levels of effector molecules. Activation of NF-kappa B by ectopic expression of IKK2 was increased in most cases by TRAF1 (Fig. 3A) – but at a higher ratio of TRAF1 compared to IKK2, we also observed a downregulation by TRAF1 (Fig. S1). When a constant amount of an IKK2-expression construct was co-transfected with increasing amounts of a TRAF1 construct, we observed an augmentation of NF-kappa B activation with saturation characteristics (Fig. 3A). Interestingly, we could detect a higher IKK2-protein expression correlating with higher levels of TRAF1 although a constant amount of IKK2-expression plasmid was applied. Calculating the degree of NF-kappa B activation normalized to IKK2 protein expression revealed a stimulatory effect of TRAF1 at lower concentrations, but an inhibitory effect at higher levels. Based on this observation, we determined the effect of TRAF1 on the expression of IKK2 mRNA and protein. Quantitative PCR analysis revealed an upregulation of IKK2-mRNA by TRAF1 (Fig. S2A) and furthermore a prolonged IKK2-protein stability as determined after blocking protein neo-synthesis with cycloheximide (Fig. S2B). We then went on to test the consequence of enforced TRAF1 expression on TNFα-mediated NF-kappa B activation. Here, we observed in most cases a dose-dependent TRAF1-mediated suppression of NF-kappa B (Fig. 3B). We suggest that the high ratio of ectopically expressed TRAF1 to endogenous IKK2 results in a predominantly inhibitory effect of TRAF1 on NF-kappa B activity as also seen in the previous transfection experiments (Fig. S1). Given that TRAF1 is an NF-kappa B dependent gene, which can be strongly upregulated by TNFα (see Fig. S3), it might act as stimulatory feedback molecule at an early phase of NF-kappa B activation, while it exerts an inhibitory function at later stages, when higher TRAF1 levels are reached. In line with the effects of TRAF1 on IKK2-mediated NF-kappa B reporter gene activity, we also observed influences of TRAF1 on IKK2 activity in kinase assays, where TRAF1 was often stimulatory (Fig. S4). However, in some cases, we also detected a slight inhibition of IKK2 activity by TRAF1 (data not shown), resembling the effects shown in reporter gene assays (Fig. S1). The influence of TRAF1 on IKK2 activity might be triggered by direct binding, but may also be the consequence of TRAF1 effects on IKK2 expression or the recruitment of activating molecules. Since TRAF2 is an important activator of the NF-kappa B-pathway interacting with TRAF1, we also tested the effect of TRAF1 on TRAF2. Upon ectopic expression as a fluorescent fusion protein in cells, TRAF2 forms distinct clusters in the cytosol. This likely reflects the inherent property of TRAF2 to trigger the oligomerization of other signaling molecules or kinases by self-interaction via the TRAF-domain. Co-expression of a CFP-tagged TRAF1 with YFP-tagged TRAF2 resulted in a dose dependent disaggregation of the TRAF2 clusters, revealing that TRAF1 has the capability of influencing the oligomerization of TRAF2 (Fig. 4) important for its signaling function.

Of note, TRAF1 is transcriptionally upregulated by NF-kappa B (Fig. S3 and [7], [10]), and degradation of TRAF2 is triggered by the NF-kappa B signaling pathway [6], [7]. The combination of these two effects alters the TRAF1/TRAF2 ratio and may therefore lead to a change from TRAF1/TRAF2 complexes to TRAF1/IKK2 complexes. Furthermore, it could also alter the composition of trimeric TRAF complexes from activating (TRAF2)_3_ or (TRAF2)_2_-TRAF1 complexes towards more inhibitory TRAF2-(TRAF1)_2_ or (TRAF1)_3_ complexes [8]. This has potential consequences for IKK2 and NF-kappa B activity dependent on the relative levels of TRAF1, TRAF2 and IKK2. Since TRAF1 does not contain a RING domain for K63-linked polyubiquitination, we expect it to be an inhibitor rather than an activator of NF-kappa B signaling by itself. However, since TRAF1 binds activators of NF-kappa B signaling such as RING domain containing TRAF molecules, it may also function as an activator in conjunction with these proteins at a certain stoichiometry. Moreover, binding of TRAF1 to IKK2 may also directly influence IKK2 activity by affecting proximity-induced auto-phosphorylation and self-activation of IKK2. Furthermore, we could show that TRAF1 influences IKK2 expression levels. Taken together, it seems likely that TRAF1 exerts variable regulatory functions in NF-kappa B signaling depending on the presence and relative levels of other signaling molecules in a given cellular context.

## Materials and Methods

### Materials

Antibodies for TRAF1 (H-125), TRAF2 (H-249), IKK2 (H-470) and HA (HA-probe Y-11) were from Santa Cruz Biotechnology Inc; antibodies against the flag-tag were from Sigma (flag-M2, F-10804) as well as flag-affinity matrix for immunoprecipitations. Antibodies against GFP were from Clontech (anti-GFP peptide antibody). IKK2 expression constructs are described in [14], [16], HA-tagged expression constructs of TRAF1 and TRAF2 have been provided by Robert Brink, a flag-tagged TRAF2 construct was provided by Tularik. CFP- and YFP-tagged constructs are described in [16].

### Cell culture and transfections

HEK-293 cells were cultured as described [17] and transfected with Lipofectamine, (Invitrogen, Carlsbad, CA, USA) or Fugene (Roche, Vienna, Austria) according to the manufacturers' protocols.

**Yeast two-hybrid screening** was performed with the C-terminal part of IKK2 (amino acids 466–756) essentially as described [14] using a library from activated leukocytes containing three million independent clones (Clontech Laboratories Inc, Mountain View, CA, USA). After identification of TRAF1 as potential binding partner, all the TRAF molecules (TRAF1-TRAF6; kindly provided by David Sassoon [18]) were tested in the yeast two-hybrid system for interaction with the IKK2 bait.

**Mammalian two-hybrid assays** were performed with the Matchmaker™ system provided by Clontech Laboratories Inc (Mountain View, CA, USA) essentially according to the instructions of the manufacturer with the exception that the pFR-Luc vector from Stratagene Inc. (La Jolla, CA, USA) was used to detect the interaction by measurement of luminescence. Full length IKK2 was cloned into the pM-vector and the prey from the yeast two-hybrid screen (TRAF1_1-228_) was cloned into the pVP16 vector.

**Coimmunoprecipitations** were carried out basically as specified in [19] using transfected HeLa or HEK-293 cells. In brief, cells were transfected with the respective expression constructs followed by lysis in detergent buffer (0.5% NP-40, 50 mM Tris–HCl pH 7.5, 1 mM EDTA, 150 mM NaCl and Complete™ protease inhibitor cocktail from Roche). Lysates were cleared by centrifugation at 4°C, 14000 rpm for 15 min. Supernatants were subject to immunoprecipitation (for 2h at 4°C) with anti-flag affinity matrix (Sigma-Aldrich, Vienna, Austria) or the respective antibodies coupled to protein A-sepharose beads (GE healthcare, Munich, Germany). Incubation of cell extracts with beads was done at 150 mM, 250 mM or 500 mM NaCl concentration to achieve different stringencies. Beads were washed four times at the respective salt concentration, incubated for 5 min at 95°C with 1x SDS-PAGE buffer and the released proteins were separated by SDS-PAGE and analyzed by Western Blotting using the indicated antibodies.

**Luciferase reporter gene assays** were performed as described in [17]. Cells were transfected with a NF-kappa B dependent Luciferase expression construct (5x NF-kappa B-Luciferase from Stratagene Inc., or a 4x NF-kappa B construct generated by inserting CTGGGACTTTCCTCTGCTGAGAAACTTTCTGCTGGGACTTTCCTCTGTCTCCGC CTGGGACTTTCCTCTGCTGAGAAACTTTCTGCTGGGACTTTCCTCTGTCTCCGC-3′ into the enhancer region of the 5x NF-kappa B vector backbone at the Apa I site). The luciferase reporter construct was transfected in combination with a constitutively expressing β-Galactosidase normalization construct (driven by a ubiquitin-promoter: pUB6/V5-His/lacZ from Invitrogen) and various IKK2 or TRAF1 expression constructs.

Cell extracts were prepared in 50 µl of lysis buffer (0.1 M KH_2_PO_4_ pH 7.8; 0.1% Triton-X100 and Complete protease inhibitor cocktail from Roche).

Luciferase activity was determined by injecting 50 µl of 1 mM luciferin in a mixture of 50 µl assay buffer (20 mM MgSO4, 4 mM ATP and 25 mM Glycyl glycine buffer pH 7.8) and 20 µl extracts and measuring the light emission for 5 sec with a Wallac 1420 VICTOR^2^-Luminometer (Perkin Elmer, Vienna, Austria). Luciferase counts were normalized to β-galactosidase activity measured with CPRG (chlorophenol red-β-d-galactopyranoside) as substrate and colorimetric detection at 570 nm.

**Kinase assays** were done as depicted in [14]. Briefly, cells were transfected with flag-tagged wildtype or mutant IKK2 in absence or presence of TRAF1, followed by extraction in kinase assay lysis buffer (20 mM Tris–HCl, pH 7.5, 150 mM NaCl, 25 mM β-glycerophosphate, 2 mM EDTA, 2 mM pyrophosphate, 1 mM orthovanadate, 1%Triton X-100, 1 mM dithiothreitol, 1 mMNaF, and protease inhibitors). Lysates were subject to immunoprecipitation of IKK2 using anti-flag affinity matrix (Sigma) and beads were washed three times with PBS and once with kinase assay buffer (20 mM Tris-HCl, pH 7.5, 20 mM β-glycerophosphate, 10 mM MgCl2, 100 µM orthovanadate, 50 mM NaCl, 1 mM DTT, 50 µM ATP, and 1 mM NaF). 1 µg GST–IκBα was added as substrate to each sample in combination with 10 µl kinase assay buffer and 10 µCi ^32^P-γ-ATP (Amersham Biosciences, UK). The beads were then incubated for 1 h at 37°C followed by addition of SDS sample buffer, heating to 95°C for 5 min and separation of proteins by SDS-PAGE. The gel was fixed with methanol/acetic acid (10% each), dried, exposed on a phosphor screen and analyzed with PhosphorImager equipment (Molecular Dynamics, Germany).

### Quantitative PCR

mRNA was extracted from HEK-293 cells by QAIGEN RNeasy Mini Kit and reverse transcribed with the RevertAidTM H Minus FIRST strand cDNA synthesis kit K1639 from Fermentas Inc. The resulting cDNA was used for quantitative realtime PCR on a StepOne Plus instrument from Applied Biosystems with SYBR green detection. Primers for TRAF1 were: forward: 5′GGAGGCATCCTTTGATGGTA′3 and reverse: 5′AGGGACAGGTGGGTCTTCTT'3; primers for IKK2 were: 5′TGCAACTGATGCTGATGT-3′ and 5′GCCTTGAAGCAGCCATT-3′; primers for the housekeeping gene human beta 2 globulin were: 5′GATGAGTATGCCTGCCGTG-3′ and 5′CAATCCAAATGCGGCATCT-3′. StepOne Plus software was used to calculate crossing threshold (Ct) points from the fluorescence curves and the delta/delta-Ct method [20] was applied to quantify the induction of mRNA as compared to the control sample. **Fluorescence microscopy** of CFP-tagged TRAF1 and YFP-tagged TRAF2 was done with a Nikon Diaphot TMD microscope as described in [16] or with a Zeiss Axiovert 135 microscope equipped with a Photometrics Coolsnap camera and appropriate fluorescence filters controlled by Metamorph 7.5 software. Fluorescence Resonance Energy Transfer (FRET) microscopy was performed by the 3-Filter method as described in [21], [22].

### Ethics Statement

This study was conducted according to the principles expressed in the Declaration of Helsinki.

## Supporting Information

Figure S1 Stimulating or inhibiting effect of TRAF1 at various ratios with IKK2. HEK-293 cells were transfected with constant amounts of NF-kappa B luciferase and β-Galactosidase reporter in combination with IKK2 and TRAF1 at different ratios as indicated. Upregulation of NF-kappa B activity by IKK2 is shown as x-fold of a negative control.
(0.18 MB TIF)

Figure S2 Upregulation of IKK2 by TRAF1. A) Effect of TRAF1 on IKK2-mRNA expression: HEK-293 cells were transfected with the indicated expression constructs and IKK2-mRNA was determined by quantitative PCR and expressed as fold of control. B) Effect of TRAF1 on IKK2 protein stability: HEK-293 cells were transfected with IKK2 alone or in combination with TRAF1. Cycloheximide was added at different time points to stop protein neo-synthesis, followed by extraction of cells and Western Blot analysis of IKK2. The IKK2 band was quantified by ImageJ analysis and expressed as percentage of the starting level.
(0.27 MB TIF)

Figure S3 Induction of TRAF1 by TNFα. HEK-293 cells were treated for different periods of time with TNFα (50 ng/ml), Upregulation of TRAF1 mRNA was determined by quantitative PCR and the induction of TRAF1 protein levels by Western Blot analysis.
(0.25 MB TIF)

Figure S4 Effect of TRAF1 on IKK2 activity. A) In vitro kinase assay using IKK2 immunoprecipitated from HEK-293 cells transfected with IKK2 alone or in combination with TRAF1 as indicated. IκBα was used as substrate and phosphorylation with 32P detected by PhosphorImager analysis. Protein levels of IKK2 were analyzed by immunoblotting (IB). B) Quantification of IκBα phosphorylation as related to the IKK2-protein level determined in A.
(0.11 MB TIF)
