# Supplementary figures and images for: Interaction of the TNFR-Receptor Associated Factor TRAF1 with I-Kappa B Kinase-2 and TRAF2 Indicates a Regulatory Function for NF-Kappa B Signaling
Source: PLoS One. 2010 Sep 13;5(9):e12683. doi: 10.1371/journal.pone.0012683 (PMC2938345; doi:10.1371/journal.pone.0012683)

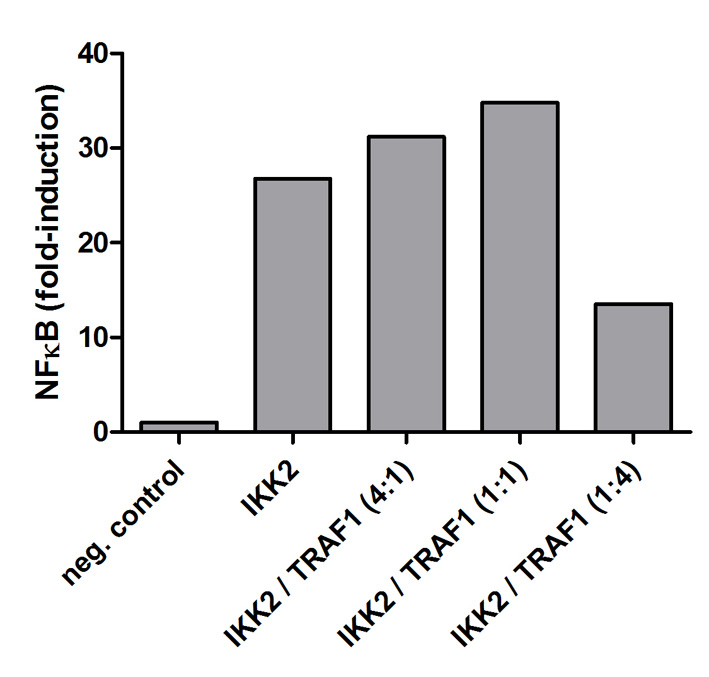

Supplement: Figure S1 — Stimulating or inhibiting effect of TRAF1 at various ratios with IKK2. HEK-293 cells were transfected with constant amounts of NF-kappa B luciferase and β-Galactosidase reporter in combination with IKK2 and TRAF1 at different ratios as indicated. Upregulation of NF-kappa B activity by IKK2 is shown as x-fold of a negative control. (0.18 MB TIF) [file pone.0012683.s001.tif]

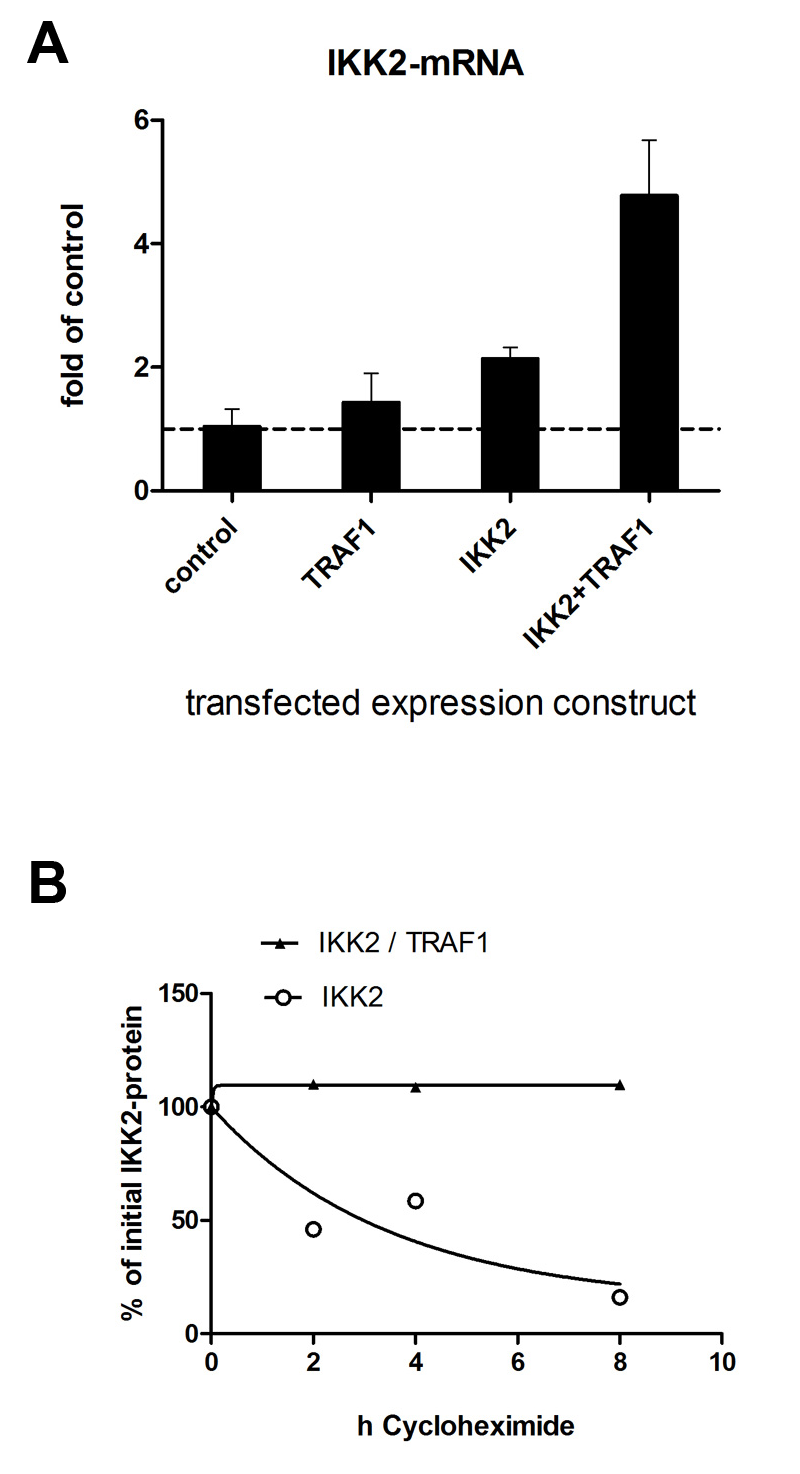

Supplement: Figure S2 — Upregulation of IKK2 by TRAF1. A) Effect of TRAF1 on IKK2-mRNA expression: HEK-293 cells were transfected with the indicated expression constructs and IKK2-mRNA was determined by quantitative PCR and expressed as fold of control. B) Effect of TRAF1 on IKK2 protein stability: HEK-293 cells were transfected with IKK2 alone or in combination with TRAF1. Cycloheximide was added at different time points to stop protein neo-synthesis, followed by extraction of cells and Western Blot analysis of IKK2. The IKK2 band was quantified by ImageJ analysis and expressed as percentage of the starting level. (0.27 MB TIF) [file pone.0012683.s002.tif]

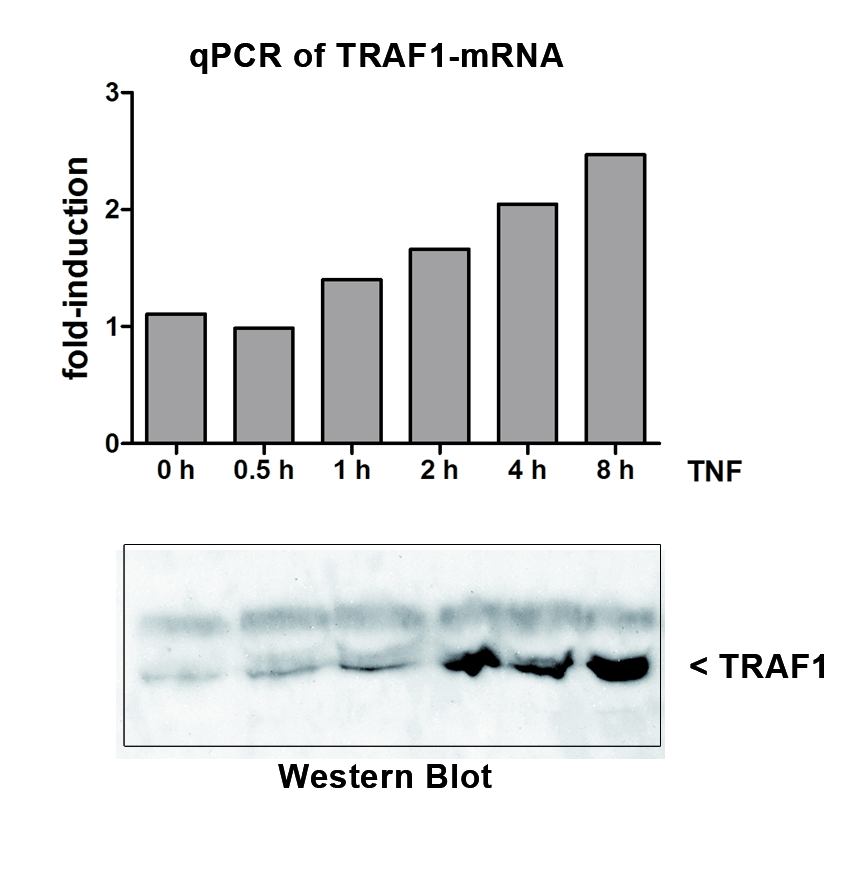

Supplement: Figure S3 — Induction of TRAF1 by TNFα. HEK-293 cells were treated for different periods of time with TNFα (50 ng/ml), Upregulation of TRAF1 mRNA was determined by quantitative PCR and the induction of TRAF1 protein levels by Western Blot analysis. (0.25 MB TIF) [file pone.0012683.s003.tif]

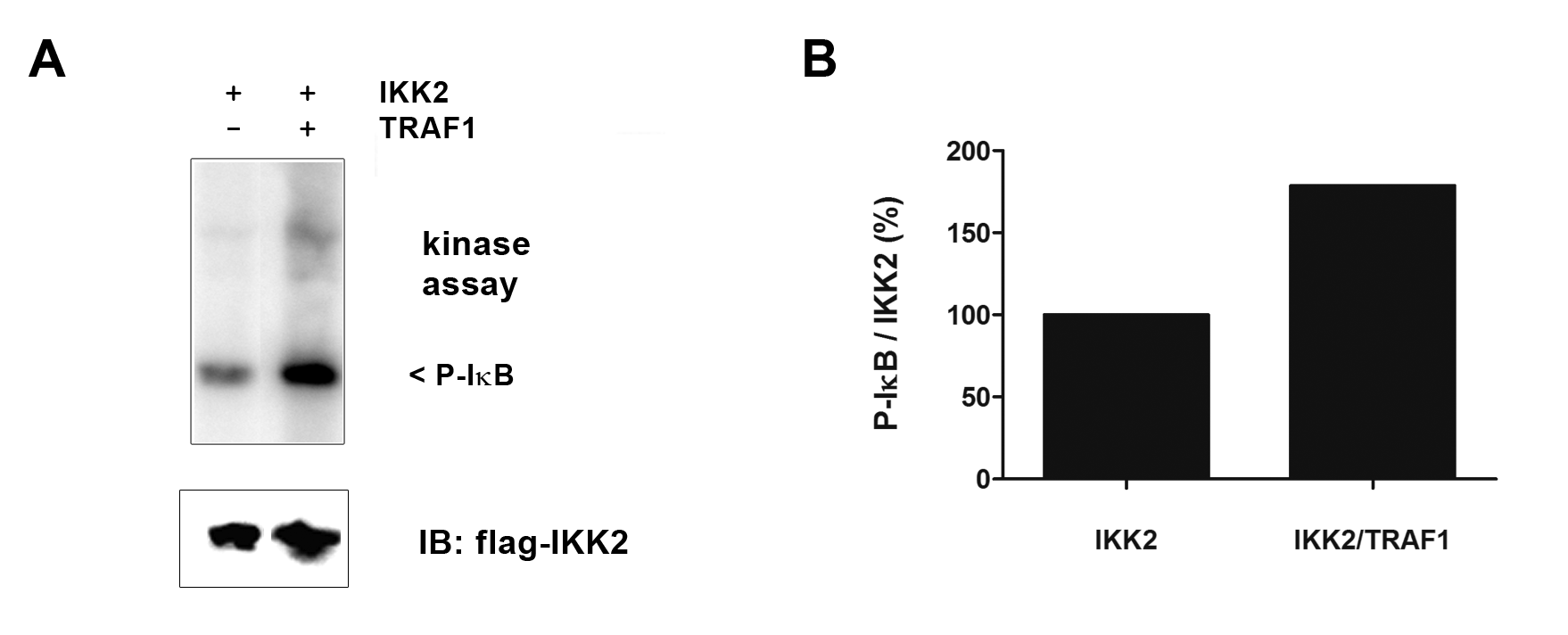

Supplement: Figure S4 — Effect of TRAF1 on IKK2 activity. A) In vitro kinase assay using IKK2 immunoprecipitated from HEK-293 cells transfected with IKK2 alone or in combination with TRAF1 as indicated. IκBα was used as substrate and phosphorylation with 32P detected by PhosphorImager analysis. Protein levels of IKK2 were analyzed by immunoblotting (IB). B) Quantification of IκBα phosphorylation as related to the IKK2-protein level determined in A. (0.11 MB TIF) [file pone.0012683.s004.tif]
